# Network Pharmacology and Molecular Docking Analysis on Pharmacological Mechanisms of *Astragalus membranaceus* in the Treatment of Gastric Ulcer

**DOI:** 10.1155/2022/9007396

**Published:** 2022-01-31

**Authors:** Piao Zhou, Rui Zhou, Yao Min, Li-Ping An, Fei Wang, Quan-Yu Du

**Affiliations:** Hospital of Chengdu University of Traditional Chinese Medicine, Chengdu, China

## Abstract

**Background:**

*Astragalus membranaceus* (AM, family: Leguminosae) exerts significant therapeutic effect on gastric ulcer (GU); however, there are scarce studies on its molecular mechanism against GU. This study aims to explore the key ingredients, key targets, and potential mechanisms of AM in the treatment of GU by utilizing network pharmacology and molecular docking.

**Methods:**

Several public databases were used to predict the targets of AM and GU, respectively, and the drug and disease targets were intersected to obtain the common targets. Next, the key ingredients and key targets were identified by constructing ingredient-target network and protein-protein-interaction (PPI) network. Gene Ontology biological processes (GOBP) and Kyoto Encyclopedia of Genes and Genomes (KEGG) pathway enrichment analysis were carried out on the common targets in order to ascertain the biological processes and signaling pathways involved. Finally, molecular docking was conducted to verify the binding affinity between the key ingredients and key targets.

**Results:**

A total of 552 predicted targets were obtained from 23 screened active ingredients, of which 203 targets were the common targets with GU. Quercetin, kaempferol, and isorhamnetin were identified as the key ingredients by constructing ingredient-target network, and TP53, AKT1, VEGFA, IL6, TNF, CASP3, and EGFR were selected as the key targets by constructing PPI network. GOBP and KEGG pathway enrichment analysis suggested that the therapeutic effect of AM on GU involved multiple biological processes and signaling pathways related to inflammation, oxidative stress, apoptosis, cell proliferation, and angiogenesis. Molecular docking validation demonstrated that all key ingredients had good binding affinity with the key targets.

**Conclusion:**

This study revealed the key ingredients, key targets, and potential mechanisms of AM against GU, and these data may provide some crucial references for subsequent research and development of drugs for treating GU.

## 1. Introduction

Gastric ulcer (GU) is a common disease in which gastric acid and pepsin self-digest the gastric mucosa due to the imbalance of defense mechanism and injury factors of gastric mucosa caused by multiple aggressive factors, including *Helicobacter pylori* (*H. pylori*) infection, overuse of nonsteroidal anti-inflammatory drugs, excessive drinking, smoking, and stress [1, 2]. Ulcers in pyloric canal may cause spasm of pyloric smooth muscle leading to pyloric obstruction, and deep ulcers involving the blood vessels of muscular layer and even serosal layer can lead to bleeding and perforation [3, 4]. At the same time, chronic GU is considered as a precancerous lesion of gastric cancer, which can provide a favorable microenvironment for tumor transformation of gastric epithelium [5, 6]. The wide application of chemicals for *H. pylori* eradication and gastric acid secretion inhibition has improved the clinical cure rate of GU, but their therapeutic effect is limited and the recurrence rate of GU is still high [1]. Furthermore, long-term use of these drugs has certain severe side effects [7, 8].

Gastrointestinal diseases are the preponderant diseases in herbal therapy. A large number of studies in human and animal models have shown that the efficacy of herbal medicines in the treatment of GU is comparable or superior to that of omeprazole and cimetidine, and herbs display fewer adverse reactions. Besides, the cost of herbal therapy in the treatment of GU is only about one-sixth of that of western medicine [9]. Therefore, herbal therapy is regarded as a valuable alternative to treat GU in humans.

*Astragalus membranaceus* (AM), also known as “Huangqi” in China, is one of the most important Chinese herbal medicines and has a medicinal history of two thousand years [10]. More than 100 ingredients have been isolated and identified from AM, mainly including flavonoids, saponins, polysaccharides, and amino acids [11]. These ingredients show a variety of biological activities *in vivo* and *in vitro*, including immunomodulatory [12], anti-inflammatory [13, 14], antiviral [15], antifatigue [16], antiaging [10], and hypoglycemic [17] effects. Traditional Chinese medicine (TCM) believes that AM has the function of invigorating spleen-qi and regenerating tissue and thus can improve gastrointestinal function and promote the healing of GU. As a result, AM is commonly used in TCM prescriptions for GU treatment, such as Buzhong Yiqi Decoction and Huangqi Jianzhong Decoction.

**Figure 1:**
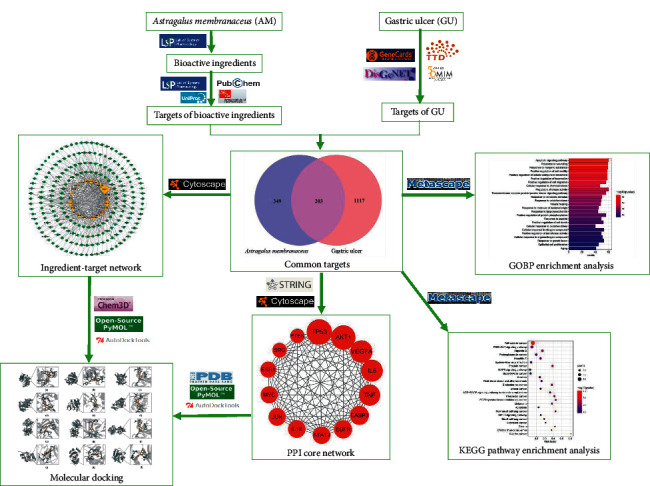
Workflow of this study.

Herbs are multi-ingredient and multitarget in treating diseases, and their pharmacodynamic ingredients and molecular mechanisms are often difficult to identify. With the advancement of network technology and bioinformatics, computer-aided identification methods of drug-target interactions represented by network pharmacology and molecular docking have been developed to provide support for the research of herbs with complex action mechanism. They greatly reduce the initial time and cost of experimental determination of drug-target interaction and improve the chances of finding ideal drug candidates [18–20]. Although AM exerts significant curative effects in the clinical treatment of GU in TCM, the relevant molecular mechanism is rarely studied at present. This study aimed to elucidate the potential mechanism of AM against GU based on the network pharmacological strategy and molecular docking, and the workflow is shown in Figure 1.

## 2. Methods

### 2.1. Bioactive Ingredients and Targets Screening of AM

The chemical ingredients of AM were obtained from Traditional Chinese Medicine Systems Pharmacology Database and Analysis Platform (TCMSP, https://tcmsp-e.com/) by using “Huangqi” as the keyword. The chemical ingredients meeting both oral bioavailability (OB) ≥30% and drug-likeness (DL) ≥0.18 were screened out as the bioactive ingredients. Besides, chemical substances that do not meet the above screening criteria but have potential activity in the treatment of GU were also included by searching and reading relevant literature on PubMed (https://pubmed.ncbi.nlm.nih.gov/).

Protein targets associated with each bioactive ingredients were acquired from TCMSP and transformed into corresponding gene symbols via Uniprot database (https://www.uniprot.org/) [21]. In addition, the SMILES strings of active ingredients were obtained from PubChem database (https://pubchem.ncbi.nlm.nih.gov/) and then were used to predict potential gene targets in SwissTargetPrediction platform (http://www.swisstargetprediction.ch/) [22, 23]. The ingredient targets obtained from two databases were merged and the redundant items were deleted.

### 2.2. Disease Targets Acquisition of GU

With “gastric ulcer” as the keyword, GeneCards Database(https://www.genecards.org/) [24], DisGeNET Database(https://www.disgenet.org/) [25], Online Mendelian Inheritance in Man (OMIM) Database (https://omim.org/) [26], and Therapeutic Target Database (TTD) (http://db.idrblab.net/ttd/) [27] were used to search and screen the known disease targets. Subsequently, all the consequences were integrated and the repeated targets were removed.

### 2.3. Ingredient-Target Network Construction

The common targets of ingredient targets and disease targets were obtained by intersection and a Venn diagram was drawn to display visually. Furthermore, a network based on the interactions between drug, ingredients, common targets, and disease was constructed and visualized by Cytoscape v3.7.1 [28].

### 2.4. PPI Network Construction

The common targets were imported into STRING database (https://www.string-db.org/) to build PPI network, and the result was exported in tab-separated value (TSV) format after hiding the unconnected nodes [29]. Subsequently, Cytoscape v3.7.1 was applied to visualize the PPI data and extract the core network according to the degree, betweenness centrality (BC), and closer centrality (CC) of nodes [28].

### 2.5. GOBP and KEGG Pathway Enrichment Analysis

The previously obtained common targets were input into Metascape (https://metascape.org/) for GOBP and KEGG pathway enrichment analysis [30]. After sorting the results according to the order of *p* value from small to large, the top 25 GOBP items and the top 25 KEGG pathways were selected to draw a bar diagram and a bubble diagram, respectively.

### 2.6. Molecular Docking Validation

In order to confirm the accuracy of the network pharmacology prediction, the key ingredients were used as small molecular ligands to perform molecular docking with the key target proteins. The 2D structures of ligands were obtained from PubChem database and structurally optimized and transformed into PDB format by Chem3D v19.0 and PyMOL v2.1.0. The 3D structures of receptor proteins were downloaded from RSCB PDB database (https://www.rcsb.org/) and converted into PDB format by PyMOL v2.1.0 after removing water and original ligands [31]. Subsequently, AutoDockTools v1.5.6 were employed for molecular docking to evaluate the binding affinity [32]. According to the principle of molecular docking, the smaller the binding energy, the more stable the docking module. The minimum binding energy <−5.0 kcal/mol indicates that a good binding affinity is between receptor and ligand, and the minimum binding energy <−7.0 kcal/mol represents that the binding affinity was extremely strong [33, 34]. Finally, the docking results with binding energy <−7.0 kcal/mol were visualized by utilizing PyMOL v2.1.0.

## 3. Results

### 3.1. Bioactive Ingredients and Targets Screening of AM

Among the 87 chemical ingredients of AM obtained from TCMSP, 20 ingredients with OB ≥30% and DL ≥0.18 were screened out as the bioactive ingredients.Astragaloside IV (OB = 22.50, DL = 0.15), astragaloside III (OB = 31.83, DL = 0.10), and ononin (OB = 11.52, DL = 0.78) were included together for their reported antiulcer potential in several studies though they did not meet the inclusion criteria [35–37]. Finally, a total of 23 active ingredients were included, as shown in Table 1, and after deleting the duplicates, 552 targets of these active ingredients were collected in all.

### 3.2. Disease Targets Acquisition of GU

1226 known disease targets of GU were collected from GeneCards Database, 136 from DisGeNET, 79 from OMIM, and3 from TTD. A total of 1320 disease targets were collected by merging the disease targets obtained from these databases and removing the duplicates.

### 3.3. Ingredient-Target Network Construction

**Figure 2:**
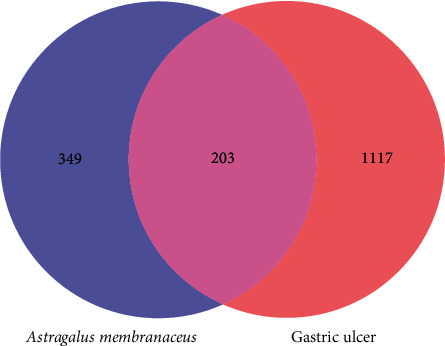
Common targets of AM and GU.

**Figure 3:**
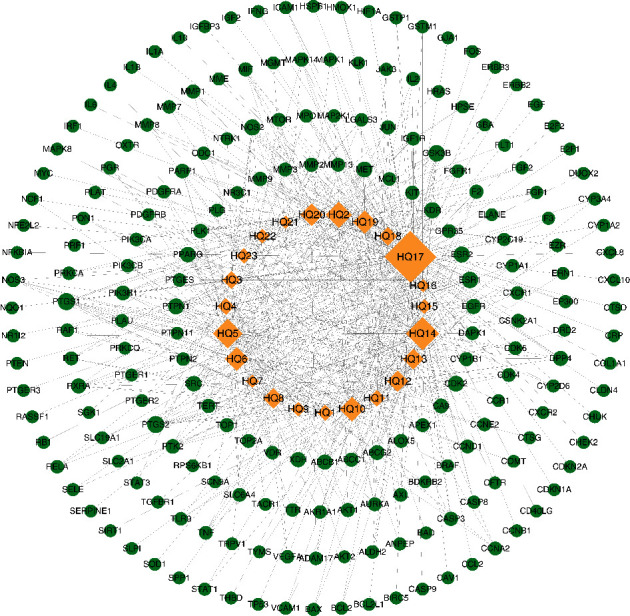
Ingredient-target network. The yellow diamonds represent bioactive ingredients, and the green circles represent the common targets. The edges represent the interaction between the ingredients and targets. Node size is proportional to its degree value. The larger the node, the more important the target in the network.

203 common targets of AM and GU were identified (Figure 2) and an ingredient-target network was constructed (Figure 3) to clarify how the bioactive ingredients of AM may act against GU [28]. The bioactive ingredients were sorted according to the descending order of degree value, and the top 3, including quercetin (degree = 123, BC = 0.5545, and CC = 0.5528), kaempferol (degree = 65, BC = 0.1186, and CC = 0.4269) and isorhamnetin (degree = 52, BC = 0.0620, and CC = 0.4039) were predicted to be the key ingredients.

### 3.4. Protein-Protein-Interaction (PPI) Network Construction

**Figure 4:**
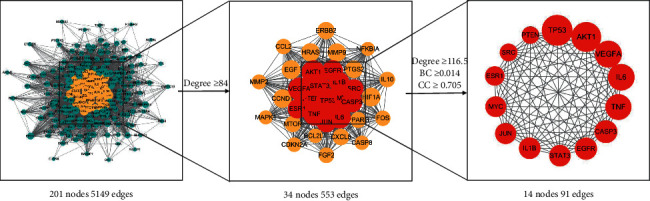
PPI core network screening flowchart. The node size is proportional to its degree value. The larger the node, the more important the target in the network.

The PPI network exported from STRING database had 201 nodes (two free nodes were hidden) and 5149 edges. The nodes with degree ≥84 were selected to build a new network, but there were still too many targets. Therefore, a core network composed of 14 nodes and 91 edges was further extracted by using the median of degree, BC, and CC. The extraction process is shown in Figure 4, and the details of core targets are listed in Table 2. TP53, AKT1, VEGFA, IL6, TNF, CASP3, and EGFR were predicted as the key targets according the descending order of degree values.

### 3.5. GOBP and KEGG Pathway Enrichment Analysis

**Figure 5:**
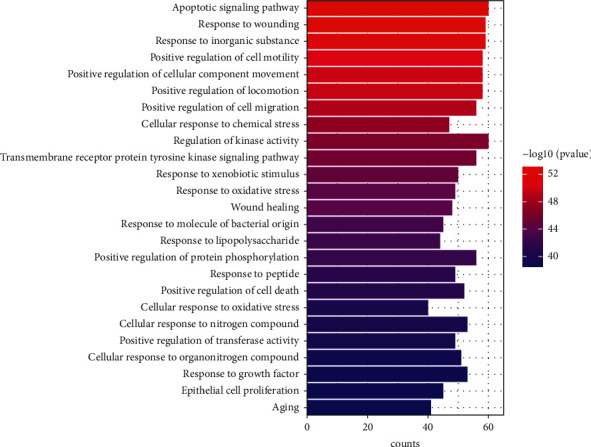
Top 25 GOBP items. The y-axis represents GOBP item. The *x*-axis indicates the number of genes enriched in the item. The redder the color, the smaller the *p* value and the more important the GOBP items.

**Figure 6:**
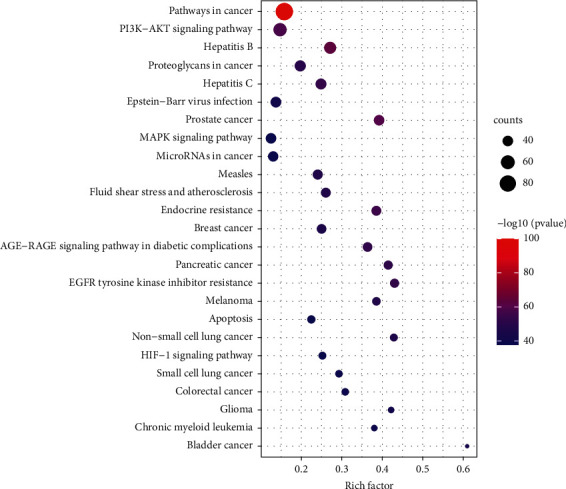
Top 25 KEGG pathways. The *y*-axis represents the KEGG pathway. The *x*-axis shows the number of genes enriched in the pathway. The size of bubbles represents the number of targets in the pathway and the color represents the *p* value. The bigger the bubble size, the more the targets in the pathway. The redder the color, the smaller the *p* value.

GOBP and KEGG pathway enrichment analysis was performed on 203 common targets using Metascape platform. According to the ascending order of *p* values, the top 25 GOBP items and the top 25 KEGG pathways were selected to draw a bar diagram (Figure 5) and a bubble diagram (Figure 6), respectively.

GOBP enrichment analysis showed the common targets involved multiple biological processes of gastric mucosa injury and repair, including apoptotic signaling pathway, cellular response to chemical stress, response to inorganic substance, response to oxidative stress, response to molecule of bacterial origin, response to wounding, response to lipopolysaccharide, wound healing, positive regulation of cell motility, positive regulation of cell migration, response to growth factor, and epithelial cell proliferation.

KEGG pathway enrichment analysis indicated that common targets were significantly enriched in pathways involving gastric mucosal injury, ulcer healing, and progression of GU to gastric cancer, such as PI3K-AKT signaling pathway, HIF-1 signaling pathway, MAPK signaling pathway, apoptosis, pathways in cancer, and MicroRNAs in cancer.

### 3.6. Molecular Docking Validation

**Figure 7:**
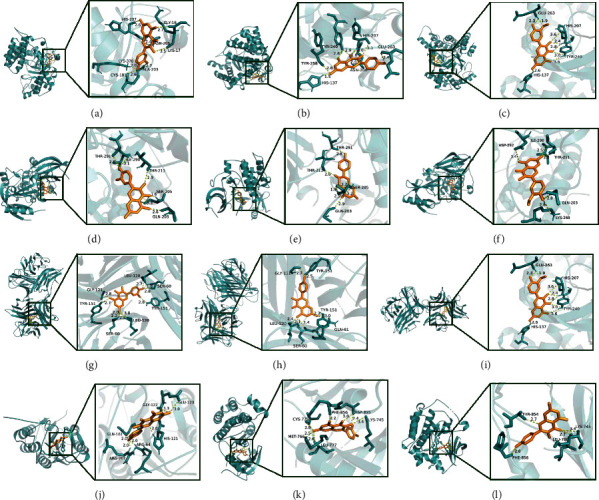
The docking modules with binding energy less than −7.0 kcal/mol. (a) TP53 with quercetin. (b) TP53 with kaempferol. (c) TP53 with isorhamnetin. (d) AKT1 with quercetin. (e) AKT1 with kaempferol. (f) AKT1 with isorhamnetin. (g) TNF-*α* with quercetin. (h) TNF-*α* with kaempferol. (i) TNF-*α* with kaempferol. (j) CASP3 with kaempferol. (k) EGFR with quercetin. (l) EGFR with kaempferol. The gold sticks represent the ligands, the cyan sticks represent the active site residues of receptor proteins, and the hydrogen bonds are represented by yellow dotted lines.

Molecular docking was conducted to evaluate the binding affinity between the 3 key targets screened from PPI network and the 7 key ingredients screened from ingredient-target network. The results verified that all key ingredients had good binding affinity with the key targets. Minimum binding energy of each docking module was recorded in Table 3, and the docking modules with minimum binding energy less than −7.0 kcal/mol are shown in Figure 7.

## 4. Discussion

In this study, the material basis and molecular mechanism of the anti-GU action of AM were explored by using network pharmacology and molecular docking. Through the construction of ingredient-target network and PPI network, three flavonoids (quercetin, kaempferol, and isorhamnetin) were predicted to be the key ingredients of AM in the treatment of GU, and TP53, AKT1, VEGFA, IL6, TNF, CASP3, and EGFR were identified as the key targets. Molecular docking also verified that all key ingredients had good binding affinity with the key targets. GOBP and KEGG pathway enrichment analysis suggested that the therapeutic effect of AM on GU involved multiple signaling pathways and biological processes related to the occurrence and healing of GU, such as inflammation, oxidative stress, apoptosis, cell proliferation, and angiogenesis.

### 4.1. AM May Improve GU via Its Antibacterial and Antiviral Activities

H. pylori is the leading cause of GU[38]. The biological activities against *H. pylori* of the screened key ingredients have been reported previously. Quercetin was found to exert excellent inhibitory activity on urease, an enzyme which enables *H. pylori* to survive in acidic gastric juice and infect gastric mucosa [39]. Kaempferol significantly reduced *H. pylori* colonies in a dose-dependent manner both *in vitro* and *in vivo* [40]. GOBP enrichment analysis also revealed that the treatment of AM on GU was related to the response to molecule of bacterial origin and response to lipopolysaccharide, suggesting that AM can alleviate gastric mucosal damage of virulence factors produced by *H. pylori.* Furthermore, KEGG pathway enrichment analysis showed that EBV infection was associated with the antiulcer effect of AM, and Epstein-Barr virus (EBV) was proved to promote gastrointestinal mucosal inflammation, indicating that AM may be considered for the treatment of GU caused by EBV [41, 42].

### 4.2. AM May Improve GU via Its Anti-Inflammatory Effect

Gastric mucosal inflammation caused by increased expression of proinflammatory factors is an important reason for the occurrence, aggravation, and long-term nonhealing of GU [43, 44]. TNF-*α* and IL-6 are regarded as the major contributors to gastric inflammation and mucosal injury, and their serum levels are positively correlated with the severity of GU [45–47]. Our study found that they were both the key targets of AM in the treatment of GU and had good binding affinity with all key ingredients. Meanwhile, evidence from existing studies has also confirmed that the screened key ingredients attenuated gastric mucosal inflammation by downregulating the expression of these two proinflammatory factors [48–50]. Furthermore, MAPK signaling pathway and PI3K-AKT signaling pathway have been already verified to ameliorate GU by regulating proinflammatory cytokines, and our study revealed that the common targets between AM and GU are enriched in these two pathways [51, 52].

### 4.3. AM May Improve GU via Its Antioxidant Effect

Oxidative stress (OS) is a state of imbalance between oxidation and antioxidation in the body, under which excessive reactive oxygen (ROS) produces oxidative damage to lipids, proteins, and DNA of cells [53]. Gastrointestinal tract is the main source of ROS, and oxidative stress plays a crucial role in gastric mucosal injury [54, 55]. Flavonoids are known as potent antioxidants, and quercetin has been proved to alleviate GU by increasing the activity of antioxidant enzymes [56, 57]. AKT is a powerful inhibitory signal of apoptosis, and the activation of PI3K-AKT pathway is crucial to reduce oxidative stress-induced damage and apoptosis of gastric mucosal cells [51]. KEGG pathway enrichment analysis revealed that the therapeutic effect of AM on GU was closely related to PI3K-AKT signaling pathway, and GOBP enrichment analysis also indicated that the antiulcer effect of AM was associated with cellular response to oxidative stress.

### 4.4. AM May Improve GU via Cell Apoptosis and Proliferation Regulation

The balance of proliferation and apoptosis of gastric epithelial cells maintains the integrity of gastric mucosa. When this balance is destroyed by various pathogenic factors and apoptosis is dominant, gastric ulcers can be induced [58]. Studies have demonstrated that quercetin promoted the normal proliferation of gastric mucosa cells through inhibiting apoptosis and cell cycle arrest induced by *H. pylori* infection [59, 60]. Among the key targets screened from PPI network, TP53, AKT1, and CASP3 are closely related to the regulation of cell cycle arrest and apoptosis, while the activation of EGFR can provide continuous intracellular division signal to induce the proliferation of epithelial cells at ulcer edge and promote their migration to the ulcer surface to repair damaged gastric mucosa [61]. GOBP and KEGG pathway enrichment analysis also suggested that the common targets were significantly enriched in the pathways and biological process involving apoptosis, response to growth factor, epithelial cell proliferation, wound healing, positive regulation of cell motility, and positive regulation of cell migration.

### 4.5. AM May Improve GU via Angiogenesis Promotion

Angiogenesis is essential for the healing of gastric mucosal injury because it contributes to delivering oxygen and nutrients to the healing site. Angiogenesis is initiated and regulated by angiogenic factors, among which VEGF is the most effective and basic regulator [62]. The fact that stimulation of angiogenesis in granulation tissues by upregulation of VEGF expression or exogenous VEGF supplementation can significantly accelerate experimental GU healing in rats indicates the important role of this target in GU treatment [63, 64]. VEGFA is one of the screened key targets of AM in the treatment of GU, and KEGG pathway enrichment analysis found some pathways in which common targets were enriched, such as PI3K-AKT signaling pathway, MAPK signaling pathway, and HIF-1 signaling pathway, can also regulate the angiogenesis of gastric mucosa through VEGF [65–67].

## 5. Conclusion

To sum up, our study has revealed that the therapeutic effect of AM on GU is multi-ingredient, multitarget, and multimechanism. The key ingredients of the curative effect are mainly flavonoids, and the molecular mechanism is closely related to inhibition of *H. pylori* and virus, anti-inflammation, antioxidation, regulation of gastric mucosal cell proliferation and apoptosis, and promotion of angiogenesis. These data can provide some crucial references for the research and development of new anti-GU drugs in the future.

## Figures and Tables

**Table 1:** Chemical properties of bioactive ingredients.

Code	Mol. ID	Molecule name	OB (%)	DL
HQ1	MOL000211	Mairin	55.38	0.78
HQ2	MOL000239	Jaranol	50.83	0.29
HQ3	MOL000296	Hederagenin	36.91	0.75
HQ4	MOL000033	(3S,8S,9S,10R,13R,14S,17R)-10,13-Dimethyl-17-[(2R,5S)-5-propan-2-yloctan-2-yl]-2,3,4,7,8,9,11,12,14,15,16,17-dodecahydro-1H-cyclopenta[a]phenanthren-3-ol	36.23	0.78
HQ5	MOL000354	Isorhamnetin	49.60	0.31
HQ6	MOL000371	3,9-Di-O-methylnissolin	53.74	0.48
HQ7	MOL000374	5′-Hydroxyiso-muronulatol-2′,5′-di-O-glucoside	41.72	0.69
HQ8	MOL000378	7-O-Methylisomucronulatol	74.69	0.30
HQ9	MOL000379	9,10-Dimethoxypterocarpan-3-O-*β*-D-glucoside	36.74	0.92
HQ10	MOL000380	(6aR,11aR)-9,10-Dimethoxy-6a,11a-dihydro-6H-benzofurano [3,2-c]chromen-3-ol	64.26	0.42
HQ11	MOL000387	Bifendate	31.10	0.67
HQ12	MOL000392	Formononetin	69.67	0.21
HQ13	MOL000417	Calycosin	47.75	0.24
HQ14	MOL000422	Kaempferol	41.88	0.24
HQ15	MOL000433	FA	68.96	0.71
HQ16	MOL000442	1,7-Dihydroxy-3,9-dimethoxy pterocarpene	39.05	0.48
HQ17	MOL000098	Quercetin	46.43	0.28
HQ18	MOL000391	Ononin	11.52	0.78
HQ19	MOL000398	Isoflavanone	109.99	0.30
HQ20	MOL000438	(3R)-3-(2-Hydroxy-3,4-dimethoxyphenyl)chroman-7-ol	67.67	0.26
HQ21	MOL000439	Isomucronulatol-7,2′-di-O-glucosiole	49.28	0.62
HQ22	MOL000405	Astragaloside III	31.83	0.10
HQ23	MOL000407	Astragaloside IV	22.50	0.15

**Table 2:** Details of 14 targets in the core network.

Number	Uniprot ID	Gene name	Protein name	Degree
1	P04637	TP53	Cellular tumor antigen *p*53	157
2	P31749	AKT1	RAC-alpha serine/threonine-protein kinase	154
3	P15692	VEGFA	Vascular endothelial growth factor A	146
4	P05231	IL6	Interleukin-6	145
5	P01375	TNF	Tumor necrosis factor	143
6	P42574	CASP3	Caspase-3	134
7	P00533	EGFR	Epidermal growth factor receptor	132
8	P40763	STAT3	Signal transducer and activator of transcription 3	130
9	P01584	IL1B	Interleukin-1 beta	130
10	P05412	JUN	Transcription factor AP-1	129
11	P01106	MYC	Myc proto-oncogene protein	128
12	P03372	ESR1	Estrogen receptor	124
13	P12931	SRC	Proto-oncogene tyrosine-protein kinase Src	119
14	P60484	PTEN	Phosphatidylinositol 3,4,5-trisphosphate 3-phosphatase and dual-specificity protein phosphatase PTEN	118

**Table 3:** Minimum binding energy between the key targets and key ingredients.

Key targets (PDB ID)	Key ingredients	Binding energy (kcal/mol)
TP53 (3TG5)	Quercetin	−8.33
	Kaempferol	−8.05
	Isorhamnetin	−8.72
AKT1 (4EJN)	Quercetin	−7.58
	Kaempferol	−7.39
	Isorhamnetin	−7.12
VEGFA (4QAF)	Quercetin	−6.07
	Kaempferol	−5.95
	Isorhamnetin	−6.53
IL6 (5FUC)	Quercetin	−5.81
	Kaempferol	−5.85
	Isorhamnetin	−5.74
TNF (6X83)	Quercetin	−7.50
	Kaempferol	−7.56
	Isorhamnetin	−7.65
CASP3 (1NME)	Quercetin	−6.85
	Kaempferol	−7.03
	Isorhamnetin	−6.61
EGFR (2RGP)	Quercetin	−7.23
	Kaempferol	−7.25
	Isorhamnetin	−6.74

## Data Availability

The figures and tables supporting the results of this study are included in the article, and the original datasets are available from the first author or corresponding author upon request.
